# New kids on the block: The Popeye domain containing (POPDC) protein family acting as a novel class of cAMP effector proteins in striated muscle

**DOI:** 10.1016/j.cellsig.2017.09.015

**Published:** 2017-12

**Authors:** Thomas Brand, Roland Schindler

**Affiliations:** Developmental Dynamics, Myocardial Function, National Heart and Lung Institute, Imperial College London, United Kingdom

**Keywords:** Popeye domain containing, cAMP signalling, Heart disease, Stress-induced bradycardia, Atrioventricular block, Limb-girdle muscular dystrophy, Protein interaction

## Abstract

The cyclic 3′,5′-adenosine monophosphate (cAMP) signalling pathway constitutes an ancient signal transduction pathway present in prokaryotes and eukaryotes. Previously, it was thought that in eukaryotes three effector proteins mediate cAMP signalling, namely protein kinase A (PKA), exchange factor directly activated by cAMP (EPAC) and the cyclic-nucleotide gated channels. However, recently a novel family of cAMP effector proteins emerged and was termed the Popeye domain containing (POPDC) family, which consists of three members POPDC1, POPDC2 and POPDC3. POPDC proteins are transmembrane proteins, which are abundantly present in striated and smooth muscle cells. POPDC proteins bind cAMP with high affinity comparable to PKA. Presently, their biochemical activity is poorly understood. However, mutational analysis in animal models as well as the disease phenotype observed in patients carrying missense mutations suggests that POPDC proteins are acting by modulating membrane trafficking of interacting proteins. In this review, we will describe the current knowledge about this gene family and also outline the apparent gaps in our understanding of their role in cAMP signalling and beyond.

## Highlights

• Popeye domain containing (POPDC) proteins are novel class of cAMP effector proteins.
• POPDC proteins control membrane trafficking of interacting proteins.
• POPDC proteins play a role in cardiac pacemaking and atrioventricular conduction.
• Mutations of POPDC genes are causing muscular dystrophy.

## 1 The cAMP signalling pathway

The second messenger cyclic adenosine 3′-5′ monophosphate (cAMP) activates one of the most important signalling pathways in the eukaryotic cell, which is involved in a large variety of cellular responses. cAMP was first discovered by Earl W. Sutherland through his work on the glycogenolytic activity of epinephrine in liver homogenates [1]. cAMP signalling involves a complex protein network, which controls second messenger production and limits diffusion resulting in compartmentalised activation of effector proteins and thereby achieving specificity.

### 1.1 β-Adrenergic receptors

**Fig. 1:**
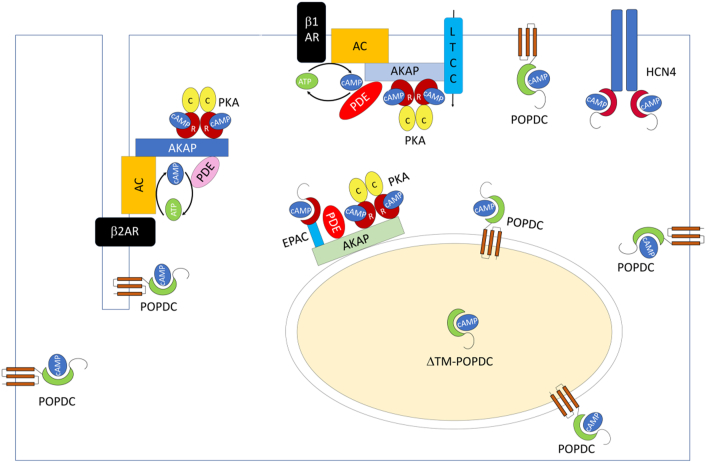
The cAMP signalling pathway in cardiac myocytes. In response to adrenergic stimulation via β1- or β2-adrenergic receptors (β1AR, β2AR), cAMP is synthesized by adenylyl cyclases (AC) and degraded by phosphodiesterases (PDEs). The effector proteins protein kinase A (PKA) and exchange protein activated by cAMP (EPAC) are part of signalosomes, which are organized by A protein kinase associated proteins (AKAPs). Target proteins, which are getting phosphorylated by PKA are often part of AKAP-dependent signalosomes. Popeye domain containing (POPDC) proteins are localized to many different subcellular compartments, some of which are also shared by other cAMP binding proteins. It is unknown whether POPDC proteins are part of the same signalosome complex as other cAMP effector proteins. Like PKA and EPAC proteins, POPDC proteins are also found in the nucleoplasm probably lacking the transmembrane domains (ΔTM-POPDC).

Activation of β-adrenergic receptors (βARs) in the heart results in a rapid rise in cAMP levels and modulate cardiac contractility (inotropy), relaxation (lusitropy), heart rate (chronotropy), conductivity (dromotropy) and cohesion (adhesiotropy) [2]. The two cardiac βAR subtypes, β1 (β1AR) and β2 (β2AR), induce a rapid rise in cellular cAMP levels, however, different cellular responses are the result of their activation. For example, sustained activation of β1AR is causing myocyte apoptosis, while β2AR activation is considered to be cardioprotective [3]. Consistent with the differing physiological responses induced by these two receptor subtypes, both display a differential subcellular distribution in ventricular myocytes (Fig. 1). Visualizing cAMP production with the help of transgenic mice, which express a fluorescent cAMP sensor in cardiac myocytes revealed that β_1_AR-stimulation induces cAMP, which propagates throughout large parts of the cell. In contrast, β_2_AR-induced cAMP synthesis is confined to transverse tubules (t-tubules), which are extensions of the sarcolemma that penetrate into the interior of striated muscle cells allowing efficient excitation-contraction coupling [4], [5].

### 1.2 Adenylate cyclases

A family of enzymes, which are termed the adenylyl cyclases (ACs), catalyze cAMP synthesis in response to activation of βARs and other hormone receptors. There are nine different membrane-bound and one soluble AC isoform [6]. In the heart, the major isoforms found in cardiac myocytes are AC5 and AC6, while AC2 and AC9 being expressed at very low levels [7]. The different AC isoforms are probably not interchangeable and the two most abundant isoforms, AC5 and AC6, are acting from opposite ends to adapt to cardiac stress. The loss of AC5 by gene targeting increases longevity and protects against cardiac stress [9] and a similar result is obtained after overexpression of AC6 [10]. Interestingly, in cardiac pacemaker cells, which are located in the sinuatrial node (SAN), the Ca^2 +^-activated isoforms AC1 and AC8 isoforms are specifically expressed along with those isoforms, which are also present in working cardiac myocytes [8]. Consistent with the preferential expression of AC8 in the SAN, forced expression causes an increase in heart rate (HR), a reduction in heart rate variability (HRV) and leads to an uncoupling of HR and HRV from autonomic control [11], [12].

### 1.3 Phosphodiesterases

The only enzymes that degrade cyclic nucleotides are the cyclic nucleotide phosphodiesterases (PDEs), which are probably the most important determinants of cAMP compartmentalization. There are 21 genes forming the PDE superfamily, which are grouped into 11 distinct PDE families, PDE1–11 [13]. All PDEs harbour a C-terminal catalytic domain of about 270 AA, which is 20–25% identical in isozymes belonging to different subfamilies. It is estimated that > 50–100 different PDE isoforms exists, which are generated by differential splicing, or through the use of alternative transcription start sites [14], [15]. Splicing generates PDE isoforms, which differ from each other in their protein interaction partners, affinity for cyclic nucleotides, reaction kinetics, regulatory mechanisms, and subcellular localization [16]. The PDEs also differ in their nucleotide specificity: PDE1–3, 10, and 11 hydrolyse both cAMP and cGMP, PDE4, 7, and 8 degrade only cAMP, and PDE5, 6, and 9 are cGMP-specific [17]. In the heart, four PDE families are responsible for cAMP degradation: PDE1 [18], [19], PDE2 [20], [21], PDE3 [22] and PDE4 [23]. The expression level of these isoforms in the heart is species-specific. In rodents, PDE4 is the major isoform, contributing up to 60% of the total cAMP hydrolytic activity, whereas PDE3 accounts for only 20 to 30% [15]. In contrast, in the human heart, PDE4 activity makes up only 10% of total PDE activity, while 90% is distributed equally between PDE1, PDE2 and PDE3 [24], [25]. Basal pacemaking in the SAN depends on PDE3 and PDE4. However, the catecholamine-induced increase in pacemaking frequency is not affected by blocking either enzyme, which suggests that the catecholamine-induced increase in pacemaking is independent of these isoforms [26]. PDE1A is expressed at high levels in the SAN [27]. PDE1A and AC1/8 are both Ca^2 +^-activated, linking cAMP production and degradation with the Ca^2 +^-clock mechanism, which together with the membrane clock is responsible for triggering cardiac pacemaking in the SAN [28].

### 1.4 Protein kinase A and AKAP proteins

The downstream targets remained unknown for a long time after cAMP has been discovered until protein kinase A (PKA) was discovered [29]. PKA is a tetramer consisting of two regulatory and two catalytic subunits. There are three isoforms of the catalytic subunit called Cα, Cβ and Cγ and a total of four regulatory subunits, RIa, RIb, RIIa and RIIb [30]. The PKA holoenzyme associates with A-kinase anchoring proteins (AKAPs), which is a large and diverse group of proteins [31]. It is estimated that there are > 30 different AKAPs, which are structurally diverse. However, a unifying principle is the presence of a 14–18 amino acids long protein kinase A binding domain, which forms an amphipathic helix mediating binding to the regulatory subunits of PKA [32]. Approximately 15 AKAPs have been identified in the heart, which play an important role in calcium-induced calcium release (AKAP18α, AKAP18 γ, AKAP79), repolarization (Yotatio, D-AKAP2) and stress-response (AKAP-Lbc, mAKAPβ, D-AKAP1) [32]. AKAPs not only bind PKA, but also other elements of the cAMP signalling pathway including other effector proteins such as exchange protein directly activated by cAMP (EPAC), ACs and PDEs and thereby forming a platform for localized cAMP generation, degradation and effector protein activation (Fig. 1). Moreover, enzymes of other signalling pathways such as calcineurin, ERK5 or PKC have been found to associate with certain AKAPs allowing cross-regulation of different signalling pathways [33], [34].

### 1.5 Exchange protein directly activated by cAMP

It was long thought that PKA and cyclic nucleotide-gated ion channels such as the hyperpolarization-activated cyclic nucleotide-gated (HCN) channels would be the only effector proteins involved in cAMP signalling. However, 1998 EPAC1 and EPAC2 were identified as novel mediators of cAMP signalling [35], [36]. Apart from having one (EPAC1) or two (EPAC2) cAMP-binding domains, EPAC proteins also have a GEF domain via which the exchange of GDP/GTP of RAP proteins is induced [37]. Both, EPAC1 and -2 are expressed in cardiac myocytes, however they are localized to different compartments. EPAC1 is mostly localized to the nuclear envelope, while EPAC2 is found along t-tubules [38]. These isoform-specific subcellular localizations agree with the proposed regulatory role of EPAC2 for ryanodine receptor (RyR) function [39] and of EPAC1 in modulating gene transcription, hypertrophy signalling and being part of a mAKAP/PKA/PDE4/ERK5 complex at the nuclear envelope [33]. EPAC1 was also described to be present at the plasma membrane and in mitochondria [40]. Recent data suggest that one of the mitochondrial functions of Epac1 is to mediate apoptotic signalling [41]. The role of EPAC1 at the plasma membrane may also include the control of membrane trafficking of the gap junction protein connexin 43 (Cx43) [42].

## 2 Discovery of the Popeye domain containing (POPDC) genes

With the goal to clone novel genes involved in heart development, two groups independently identified the first member of the Popeye domain containing (POPDC) gene family [43], [44]. Based on sequence homology, two additional members of the gene family were subsequently identified. In vertebrates, the POPDC family consists of *Popdc1* (also known as *Bves*), *Popdc2* and *Popdc3* [44], [45]. POPDC genes are abundantly expressed in the heart and skeletal muscle [44]. Apart from striated muscle tissue, POPDC family members are also expressed in smooth muscle tissue in different organs (lung, gastrointestinal tract, bladder, uterus), epithelial cells (skin, cornea, esophagus, pyloric epithelium), and neurons of the autonomous and central nervous system [44], [46], [47]. In the adult heart, expression of *Popdc1* and *Popdc2* is confined to cardiac myocytes and is absent from non-muscle cell type [48]. *Popdc1* and *Popdc2* are differentially expressed in different parts of the heart. In the adult heart, *Popdc1* is expressed at higher levels in the atria than the ventricles and expression is also elevated in the cardiac conduction system (CCS) which includes the SAN, the atrioventricular node (AVN), the His bundle, the bundle branches and the Purkinje fibres. *Popdc2* on the other hand is expressed at equal levels in atria and ventricles but also displays a higher expression level in the CCS [48]. A detailed expression pattern of *Popdc3* is currently not available.

### 2.1 POPDC proteins

#### 2.1.1 Protein structure

**Fig. 2:**
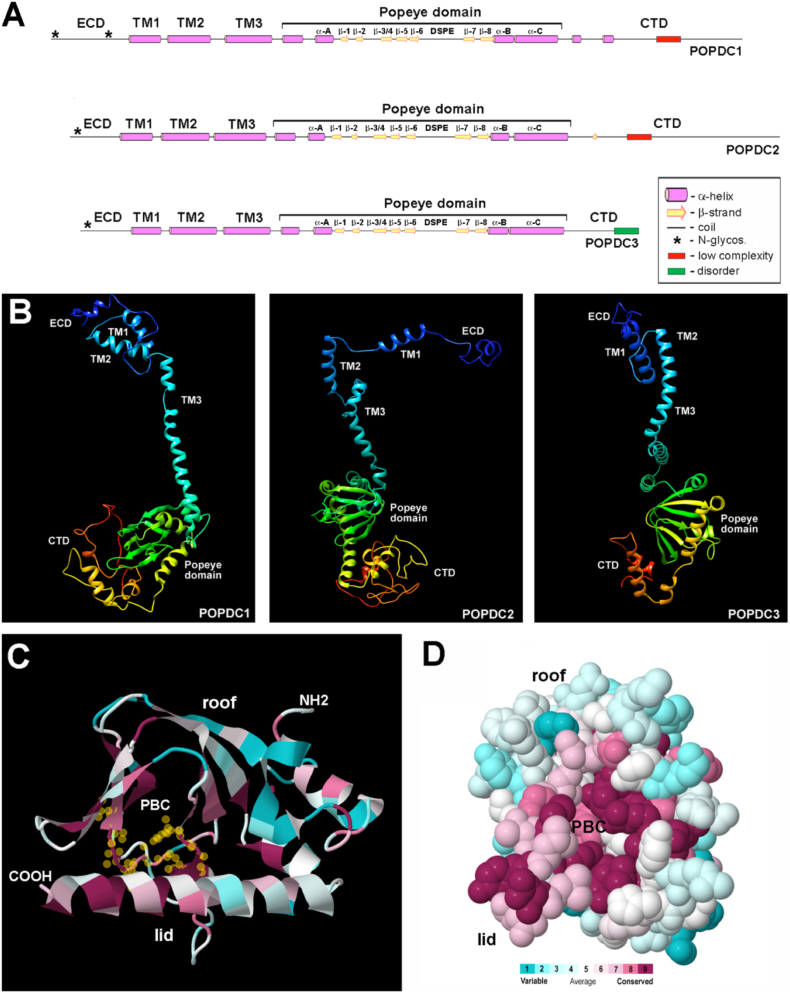
Structural modelling of the Popeye domain containing proteins. (A) Secondary structure prediction of POPDC proteins using Psipred [108]. In each isoform, a short (26–47 residues) extracellular domain (ECD) is present, which harbors one or two N-glycosylation motifs (asterisks) followed by three transmembrane domains (TM1–3). A large part of the cytoplasmic domain consists of the Popeye domain, which is close to 50% similar between family members. The carboxy-terminal domain (CTD) is most variable part of the POPDC proteins and distinct between isoforms. (B) 3-D models of POPDC1, POPDC2, and POPDC3. The models were produced with the help of the Phyre 2 web portal, which predicts 3-D protein structures on the basis of homology modelling [109]. The structures were visualized using the CCP4 molecular graphics program [110]. (C,D) Demonstration of evolutionary conservation of the Popeye domain with the help of the Consurf program [111]. (C) Cartoon and (D) Spacefill model of the Popeye domain of human POPDC1. Many invariant amino acids cluster around the PBC or are part of the lid, which is predicted to fold over the PBC when ligand is bound [98].

POPDC genes encode medium-sized transmembrane proteins (human POPDC1: 360 amino acids, predicted MW: 41.5 kDal; human POPDC2: 364 amino acids, predicted MW: 40.4 kDal; human POPDC3: 291 amino acids, predicted MW: 33.8 kDal) (Fig. 2). The amino terminus, of POPDC proteins is short and consists of only 27–39 residues. One or two N-glycosylation sites have been mapped and appear to be functional [49]. Glycosylation of POPDC proteins is extensive and the apparent molecular weight of the major form of POPDC1 in the heart is approximately 55–58 kDal [44]. Interestingly, the apparent molecular weight of POPDC1 is organ-specific and is approximately 70 kDal in skeletal muscle [50]. POPDC proteins have three transmembrane domains. In the cytoplasmic part of the protein is the Popeye domain (Pfam domain: PF04831) localized (Fig. 2). The Popeye domain is a 150 amino acid long, evolutionary conserved protein domain, which displays a strong structural similarity to the cAMP binding domains of the regulatory subunit of PKA [48]. However, sequence similarity to the cAMP binding domains of PKA or EPAC is very low. While the Popeye domain is unique and only found in POPDC proteins, non-POPDC proteins with the highest sequence similarity are the catabolite activator proteins (CAPs), also known as cAMP receptor proteins (CRPs). This family of transcription factors, which are exclusively found in bacteria, are involved in metabolic control and cyclic AMP functions as an allosteric effector by increasing the affinity of CRP or CAP for DNA [51]. In this regard, it is noteworthy that for example in satellite cells, POPDC proteins are present in the nucleoplasm [52]. Nuclear localization appears to be regulated by cell differentiation and is lost after myotube formation. It will be interesting to investigate, whether the nuclear-localized POPDC isoforms also have gene regulatory functions.

#### 2.1.2 Cyclic nucleotide binding properties

Two invariant sequence motifs (FL/I**DSPE**W/F and **FQVT**/SL/I) in the putative binding pocket of the Popeye domain are linked by a non-conserved sequence, which is variable in length in different Popeye domains. Both, the DSPE and FQVT motifs are crucial for cAMP binding and are strongly conserved in evolution (Fig. 2). Charge-to-alanine mutations of D200 of POPDC1 and the homologous residue D184 of POPDC2 strongly impair cAMP binding [48]. Mutation of the ultra-conserved serine 201 to phenylalanine also led to an impairment of cAMP binding of POPDC1 as did the Glu203 to Ala and Val217 to Phe mutations [53]. Measurement of the cyclic nucleotide affinity with the help of recombinant POPDC protein in a competitive radioligand binding assay, lead to an estimate of the cyclic nucleotide binding affinity, which is approximately 118 nM for cAMP and approximately 40-fold lower (5 μM) for cGMP. Similar affinity data were independently obtained with the help of a bimolecular Förster-resonance energy transfer (FRET) assay [48]. The carboxy-terminal domain (CTD) of POPDC proteins is variable in sequence and length and subject to alternative splicing. While being divergent between POPDC family members, the CTD displays isoform-specific conservation. Proteomic analysis suggests, that the CTD of each POPDC isoform is subject to β1AR-dependent phosphorylation, which may be important in modulating the biological activity of POPDC proteins [54]. Interestingly, POPDC isoforms differ in the number of residues, which are getting phosphorylated in response to β1AR activation. POPDC1 appears to be the most targeted isoform with a total of 9 phosphorylation sites, POPDC2 has four sites and POPDC3 contains a single residue, which is phosphorylated in response to adrenergic agonists [54].

### 2.2 Defining POPDC function in animal models

#### 2.2.1 *Drosophila*

Loss–of-function experiments have been performed in a number of organisms. In *Drosophila*, a single POPDC gene (*bves*) is present in the genome. Due to the complexity of the genomic region, P-element mobilization did not result in the identification of a null mutant for *bves* [55]. As an alternative approach, heat shock-induced expression of an antisense transcript caused early lethality with embryos displaying abnormal gastrulation movements and aberrant gut closure. No further analysis of organ formation and in particular no analysis of heart and skeletal muscle structure and function has been performed in the transgenic flies.

#### 2.2.2 Zebrafish

Morpholino-mediated knockdown of *popdc2* in zebrafish caused a severe muscular dystrophy phenotype, which is characterized by the disruption of the myotendinous junction [56]. A similar phenotype was observed in case of *popdc1* morphants and in the *popdc1*^S201F^ knock-in (KI) mutant [53]. In the heart, many *popdc1* morphants and a sizable fraction of the *popdc1*^*S201F*^ KI mutant displayed a pericardial effusion and a similar phenotype was also observed in case of *popdc2* [45], [56]. While a pericardial effusion is often interpreted as a sign of myocardial pumping deficiency, an alternative interpretation was recently provided [57]. POPDC proteins have an important function in organizing tight junction formation and the lack of *popdc1* in zebrafish by morpholino-mediated knockdown causes a defective barrier formation of the skin leading to a fragility of the embryo when challenged by hyperosmotic conditions. The inability to maintain osmotic homeostasis is probably the reason for the pericardial effusion phenotype, as it was rescued by raising the osmolality of the culture medium [57]. The molecular basis for the osmotic homeostasis phenotype in *popdc1* morphants probably relates to an impaired accumulation of tight junction proteins (claudin) in the epithelium, which is caused by an aberrant localization of atypical protein kinase C (aPKC) [57]. Support for the role of POPDC1 in cell-cell contact formation stems from the observation that another important protein, which directly or indirectly interacts with POPDC1 is zonula occludens 1 (ZO1) [58]. Loss-of-function experiments in an epithelial cell line caused a loss of ZO1 from the cell surface and reduced trans‑epithelial resistance, suggesting an important function of POPDC1 in tight junction formation in epithelial cells.

Apart from the osmotic instability leading to a pericardial effusion phenotype in *popdc1* and *popdc2* morphants and *popdc1*^S201F^ mutants, also a cardiac arrhythmia phenotype was present in each of these zebrafish models [45], [56]. The first arrhythmia phenotype seen is an AV-block, which is already present at 2–3 days post-fertilization (dpf) in *popdc1* mutants and develops slightly later in *popdc2* morphants. At 5 dpf close to 40% of the *popdc2* morphants displayed a cardiac arrhythmia phenotype [45], [56]. The *popdc1*^S201F^ mutant displayed a similar phenotype as the *popdc1* morphant, however in this case the frequency of animals displaying an arrhythmia phenotype was much lower and amounted to only 5%. However, the number of animals displaying a phenotype could be tripled when the animals were challenged by isoproterenol treatment [45].

#### 2.2.3 *Xenopus*

Loss-of-function experiments of *POPDC1* using morpholinos were also performed in *Xenopus* and in contrast to what has been observed in zebrafish and in the mouse (see below) an early gastrulation phenotype was observed [59]. It is possible that differences in the expression pattern of POPDC family members between model systems may be responsible for the differences in timing of phenotype development. Interestingly, the early gastrulation phenotype in *Xenopus* is similar to what has been described in *Drosophila* [55].

#### 2.2.4 Mouse

*Popdc1* and *Popdc2* null mutants in mice were engineered by homologous recombination. While both null mutants were viable and survived into adulthood, specific pathologies were observed, when the animals were challenged. *Popdc1* null mutants displayed a regeneration phenotype in skeletal muscle after cardiotoxin injection [60]. The regeneration was retarded relative to wildtype skeletal muscle, however the underlying molecular mechanisms are currently unknown.

Induction of ischemia/reperfusion injury to Langendorff-perfused hearts caused a significantly lower functional recovery in the *Popdc1* null mutant compared with wild type, while infarct size was larger [61]. Isolated cardiac myocytes from *Popdc1* null mutants displayed impaired Ca^+ 2^-transients, increased vulnerability to oxidative stress and absence of pharmacologic preconditioning. Further studies using myocytes treated with *Popdc1* siRNA revealed that *Popdc1* is required for cardiac myocyte survival [62]. Loss of *Popdc1* causes an increase in the expression of the proapoptotic BCL2 interacting protein 3 (*Bnip3*), which may explain the increased vulnerability when null mutant hearts are subjected to ischemia/reperfusion injury.

As mentioned before, *Popdc1* and *Popdc2* display high-level expression in the CCS. Implantation of telemetric ECG devices revealed normal heart rate and a normal electrocardiogram at baseline. However, when mouse mutants were subjected to physical exercise, mental stress or isoproterenol injection, the heart rate became highly variable and the SAN pacemaker was pausing for different lengths of time [48]. Interestingly, the pathological phenotype, which is present in both *Popdc1* and *Popdc2* null mutants was not present in young mice, however, at 5–8 months of age, mutants displayed a severe stress-induced bradycardia with episodes of sinus node dysfunction. The age-dependency of phenotype development in the mutants is reminiscent of the sick sinus syndrome (SSS), which is the most frequent reason for pacemaker implantation and most prevalent in the elderly in the absence of any other heart disease [63].

### 2.3 Association of *POPDC1* mutations with heart disease

While the association of heart and muscle phenotypes in animal models carrying loss-of-function mutations was demonstrated in several species, the question, whether POPDC mutations are also disease-causing in patients remained unanswered until recently.

#### 2.3.1 Mutation in *POPDC1* causes muscular dystrophy and cardiac arrhythmia

A family of Albanian origin living in a remote location in the southern part of Italy was found to carry a recessive missense mutation in *POPDC1* (S201F) and to suffer from limb-girdle muscular dystrophy (LGMD) and type II atrioventricular block in individuals, which carried the mutation in homozygosis [45]. LGMD is a heterogeneous group of muscle diseases, which is caused by mutations in a large number of different genes. However, many LGMD-causing mutations affect proteins localized to the plasma membrane such as dystroglycan, sarcoglycans, caveolin-3 (CAV3), and dysferlin (DYSF) or the nuclear envelope (lamin A/C (LMNA)) [64]. While several LGMDs only manifest in skeletal muscle, others also involve the heart and cause arrhythmias or DCM phenotypes [65].

The missense mutation in *POPDC1* discovered in the Italian family substitutes serine 201 by phenylalanine. Serine 201 is invariant and is part of the DSPE motif, which is thought to be directly involved in cyclic nucleotide binding [48]. Substituting serine by the bulky and hydrophobic amino acid phenylalanine is likely to interfere with cAMP gaining access to its binding pocket. Measurements of cAMP affinity revealed a drop in cAMP affinity by approximately 50% [48]. Significantly, zebrafish mutants carrying the homologous mutation *popdc1*^*S191F*^ displayed both, heart and skeletal muscle pathologies, which were reminiscent of the patient's phenotypes [45]. Most importantly, the impaired nucleotide binding affected the membrane localization of the mutant POPDC1 protein. In the patient's skeletal muscle biopsies as well as skeletal muscle tissue of zebrafish mutants carrying the homologous mutation, the majority of the mutant POPDC1 protein was mislocalized and no longer present at the plasma membrane but in a peri-nuclear domain. Surprisingly, also the subcellular localization of POPDC2 was abnormal. The POPDC1^S201F^ mutation is rare and sequencing of an additional 104 patients with similar clinical phenotype did not reveal any additional carrier [48].

#### 2.3.2 Putative association *POPDC1* with other forms of cardiac disease

An association of *POPDC1* with other forms of cardiac arrhythmia was recently predicted by two studies using bioinformatics, which suggests that *POPDC1* might act as a hub gene for atrial fibrillation (AF) [66] and as a novel determinant of the length of the QT interval, which is the time between the start of depolarisation and the end of repolarisation of the ventricular chambers [67]. An increase in the QT time raises the risk of developing severe forms of ventricular arrhythmia. A down-regulation of *POPDC1* and *POPDC3*, and to a lesser degree *POPDC2* in heart failure has also been recently reported [68]. *POPDC1* variants were identified in patients with Tetralogy of Fallot (ToF) an important form of congenital heart disease (CHD) [69]. However, the discovered variants haven't been introduced into animal models to confirm that they are disease-causing. The association of *POPDC1* with ToF is therefore rather uncertain given the lack of CHD in the *Popdc1* null mutant in mice.

### 2.4 Association of POPDC genes with cancer

Apart from their association with heart and skeletal muscle disease, a significant body of knowledge has accumulated in recent years, which indicates a strong association of a loss of POPDC gene function with various cancer types (reviewed in [70]. Suppression of *POPDC1* or *POPDC3* expression correlates strongly with disease progression and poor clinical prognosis in different cancer forms including gastric cancer [71], [72], lung cancer [73], hepatocellular carcinoma [74], breast cancer [75] and colorectal cancer [76]. The reduction in *Popdc1* expression in the tumour is probably due to an increase in methylation [71], [76]. An investigation of pan-cancer hypermethylated CpG sites identified an enhancer, which is hypermethylated in at least 11 different cancer forms and is predicted to regulate *POPDC1* expression [77]. In most cases it is unclear how a reduction in POPDC expression affects tumour formation. Several changes in protein expression including an increase in WNT signalling have been noted [78]. But whether POPDC proteins are directly involved is presently unclear (see for a discussion [70], [79]). Recent work on a model of inflammation-induced colon carcinoma formation has lead to the discovery that POPDC1 controls c-Myc levels by forming a complex with the PR61α and c-Myc [78]. PR61α is a regulatory subunit of protein phosphatase 2A (PP2A), which apart from other activities promotes c-Myc degradation by dephosphorylation of a critical residue. In agreement with this finding is the observation that a knockdown of *Popdc1* caused increased c-Myc levels, whereas forced expression suppressed c-Myc.

## 3 POPDC interacting proteins in striated muscle

### 3.1 *Popdc1* is a multi-compartment protein

POPDC proteins in striated muscle cells are localized to the plasma membrane and its derivatives in striated muscle including intercalated discs [80], t-tubules and caveolae [61]. In addition, POPDC1 and POPDC2 are also present at the nuclear envelope and nucleoplasm [52], [81]. Presently, it is unknown, whether the structure of any of these membrane compartments is affected in *Popdc1* or *Popdc2* null mutants with the exception of caveolae, which were described in the heart of *Popdc1* null mutants to be reduced in number but larger in diameter [61]. Interestingly, ultrastructural analysis of skeletal muscle biopsies of one of the patients carrying the POPDC1^S201F^ mutation revealed the presence of membrane discontinuities, which were similar to those observed in case of patients carrying anoctamine-5 (*ANO5*) mutations and possibly is an indication for impaired repair of the muscle plasma membrane [82]. Consistent with these ultrastructural changes in patients and mouse mutants is the recent findings that POPDC proteins interact with Caveolin (CAV3) and Dysferlin (DYSF) [45], [61]. Interestingly, POPDC1, ANO5, CAV3 and DYSF have all been genetically linked to LGMD suggesting that they possibly all are active in the same disease pathway [64]. Membrane instability of the mutant muscle may also be related to Dystrophin, which is also an interaction partner of POPDC proteins [45].

### 3.2 *Popdc1* interacts with the two-pore potassium channel TREK-1

The plasma membrane localization of POPDC proteins, the pacemaker phenotype in *Popdc1* and *Popdc2* null mutants, the AV-block in patients and in zebrafish mutants and the high expression levels of POPDC proteins in the cardiac conduction system, all point to a role of POPDC proteins in cardiac action potential generation and/or cardiac conduction. The interaction of POPDC proteins with ion channels or other electrogenic proteins was recently tested, which resulted in the identification of the potassium channel TWIK-related K^+^ channel 1 (TREK-1) as a POPDC interacting protein [48]. TREK-1 is a member of the two-pore domain potassium channel (K_2_P) family, which is modulated by a large number of different stimuli including stretch, pH, temperature, phosphorylation, and interacting proteins [83]. In *Xenopus* oocytes, co-expression of any POPDC isoform and TREK-1 stimulates a twofold higher current [48], which is thought to be due to an enhanced membrane transport of the channel protein. This effect was modulated by cAMP and was lost in the presence of the general PDE inhibitor theophylline. The interaction of POPDC proteins with TREK-1 is mediated by the cytoplasmic part of the protein. Based on the interaction of POPDC and TREK-1, a bi-molecular FRET sensor was constructed. The FRET ratio obtained at baseline decreased after the addition of isoproterenol or forskolin, suggesting that cyclic nucleotide-binding affects the interaction of POPDC1 with TREK-1 [48]. A cardiac specific knockout of *Kcnk2*, which encodes TREK-1, displays a stress-induced sinus bradycardia similar to the one observed in *Popdc1* and *Popdc2* null mutants [84], suggesting that the sinus bradycardia in POPDC mutants may in part be due to an impaired TREK-1 current.

### 3.3 *POPDC1* might control membrane trafficking of interacting proteins

In support of a role of POPDC proteins in regulating membrane trafficking is the recent finding that the POPDC1^S201F^ mutant protein displayed a reduction in membrane localization and at the same time an increased perinuclear expression [45]. Similar to the POPDC1^S201F^ protein, POPDC2 was also mislocalized in the mutant muscle. Surprisingly, increase of cAMP levels caused a reduction in membrane localization of TREK-1 in the presence of POPDC1^S201F^, whereas a reduction in the cAMP-binding affinity of POPDC1^S201F^ caused a reduction in membrane transport of itself and of POPDC2. These data suggest that the effect of cAMP on membrane trafficking of POPDC1 and associated proteins is complex. Therefore, more work is required to unravel how cAMP-binding of POPDC proteins affect protein-protein interaction and membrane trafficking.

### 3.4 *POPDC1* interacting proteins in epithelial cells

A number of additional POPDC1 interacting proteins, which play in particular a role in epithelial cells, were recently described and include Zona occludens 1 (ZO1), guanine nucleotide exchange factor T (GEFT), vesicle-associated membrane protein 3 (VAMP3) and N-Myc downstream regulated gene 4 (NDRG4) [85], [86]. The PDZ protein Zonula occludens-1 (ZO1) [58] functions as a scaffolding protein and interacts for example with tight junction proteins. Loss of POPDC1 in cultured epithelial cells reduces transepithelial resistance and junctional protein localization at the plasma membrane [58]. In cardiac myocytes, ZO1 binds the carboxy terminus of Cx43 and thereby controls gap junction plaque size [87]. It is possible, that POPDC1 and ZO-1 are important for myocardial conduction. In this regard it is noteworthy that POPDC1 and POPDC2 are expressed in the intercalated disk [80]. VAMP3 is a soluble N-ethylmaleimide-sensitive factor attachment receptor (SNARE) protein and part of the vesicle docking and fusion complex [88]. Interestingly, VAMP proteins are involved in membrane trafficking of transmembrane proteins by modulating the recycling endosome compartment. Thus, the impaired membrane trafficking in POPDC mutants may involve SNARE protein-mediated membrane transport. POPDC1 interacts with NDRG4 and the knockdown of both genes affects membrane docking of VAMP3-labeled vesicles [86]. GEFT, a guanine nucleotide exchange factor (GEF) docking for the Rho-family GTPases Rac1 and Cdc42 is another interaction partner of POPDC1 [89]. GEFT is expressed in cardiac and skeletal muscle, however, the interaction of POPDC1 and GEFT is largely unexplored.

## 4 Regulation of POPDC gene expression

### 4.1 *Popdc1* expression is suppressed by EGF signalling

Little is known about the regulation of POPDC gene expression. In *Drosophila, bves* is suppressed in dorso-anterior follicle cells by the secretion of gurken, an epidermal growth factor (EGF)-like signalling molecule [55]. Regulation of POPDC expression by EGF was also investigated in the gastric adenocarcinoma cell line SNU-216, which expresses all three POPDC genes [71] and in several breast cancer cell lines [75]. Addition of EGF causes a repression of POPDC genes. However, POPDC2 differs from POPDC1 and -3 in kinetic and extent of suppression. Interestingly, also in cardiac myocytes, POPDC gene expression is regulated by EGF signalling [90]. Expression of all three vertebrate POPDC genes is reduced, when cardiac myocytes were cultured in the presence of serum, an effect that was rescued by the EGF receptor antagonist tyrphostin. The EGF-like signalling molecule neuregulin is secreted by endocardial cells and responsible for the formation of the trabecular layer in the developing ventricle [91], [92]. Neuregulin signalling suppresses apico-basal polarity and thereby myocytes destined to participate in trabecular layer formation acquire a mesenchymal-like state and leave the myocardial wall by a process reminiscent of EMT in epithelia. Possibly neuregulin signalling is responsible for the low level of Popdc1 expression in the trabecular layer in the midgestation mouse heart [44], [60].

### 4.2 *Popdc1* expression is suppressed by Netrin

Netrin-1, a laminin-related neuronal guidance molecule, which is also expressed in non-neural cell types, is a negative regulator of *POPDC1* gene expression [93]. Netrin-1 modulates *POPDC1* expression through PI3-kinase/AKT signalling and a specific antagonist of this signalling pathway is able to re-establish *POPDC1* expression in the presence of Netrin-1. In several cancer types, gain of Netrin 1 expression has been observed, which may lead to tumour progression and possibly is associated with the suppression of *POPDC1* expression.

### 4.3 Transcriptional regulation of POPDC genes

Apart from methylation, little is known about transcriptional regulation of POPDC gene expression. No systematic study of the regulatory elements involved in the transcriptional regulation of *Popdc1* and *Popdc3* expression has been performed. However, it has been proposed that *Popdc1* might be a target of Pax3 [94]. Popdc2 is the isoform with highest level of expression in the heart [44]. The promoter of the *Popdc2* gene has recently been characterized and found to contain a binding site for the NK homeobox protein 2.5 (Nkx2.5), which overlaps with a binding site for Meis homeobox protein 1 (Meis1) [95]. The Nkx2.5 binding site is part of a compact promoter, which is sufficient to drive cardiac expression in zebrafish and mouse embryos. Chromatin immunoprecipitation experiments using the mouse *Popdc2* promoter revealed that Nkx2.5 occupied the binding site in differentiated cardiac myocytes, whereas Meis1 is present in anterior heart field mesoderm [95].

## 5 Working models of POPDC function

### 5.1 The switch model

**Fig. 3:**
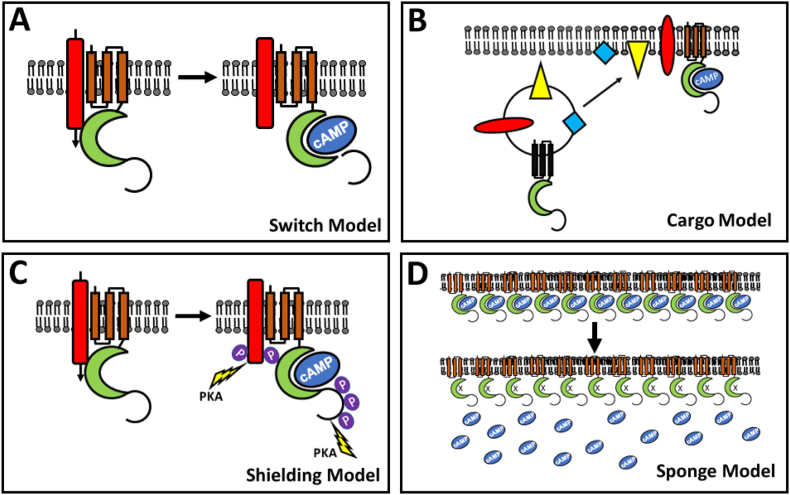
Different working models of POPDC protein function. (A) Switch model. (B) Cargo model. (C) Shielding model. (D) Sponge model. See text for a further description and the experimental evidence supporting these different models of POPDC protein function.

Over the years several working models have been proposed to describe the functions of POPDC proteins. The first model that was proposed is the *switch model* (Fig. 3A) [96], [97]. This model proposes that interacting proteins are forming a complex with POPDC proteins. Such protein complexes could be demonstrated by a number of techniques such as pull-down, immunoprecipitation or FRET experiments and one such protein is TREK1 for which interaction with POPDC1 has been demonstrated by each of the aforementioned techniques [48]. Upon binding of cAMP, it is likely that the Popeye domain alters its conformation as has been reported for all other cAMP binding proteins [98]. Cyclic nucleotide binding may affect protein-protein interaction, which may also have an impact on the functionality of the interacting protein. Support for this model comes from FRET experiments using POPDC1 and TREK-1 [48]. The POPDC1/TREK-1 FRET signal is modulated upon raising cAMP levels. However, direct measurements of TREK-1 current in *Xenopus* oocytes, which were acutely stimulated with 8-Br-cAMP did not show any difference between agonist versus vehicle-treated oocytes [45]. This negative outcome does not rule out the possibility that other proteins might be directly affected by the binding of cAMP to POPDC proteins.

### 5.2 The cargo model

The *cargo* model (Fig. 3B) describes a role of POPDC proteins in membrane trafficking of interacting proteins and this model is based on the finding that the membrane localization of TREK-1 in *Xenopus* oocytes is doubled if any of the three POPDC isoforms are present. Moreover, in patients carrying the POPDC1^S201F^ mutation and the corresponding zebrafish mutant display aberrant membrane localization of the mutant protein but also of POPDC2. POPDC proteins are not only found at the plasma membrane but are also present in vesicular structures in the sarcoplasm. While these vesicles haven't yet been further characterized, it can be envisioned that they are part of the recycling vesicle compartment. Possibly, cAMP alters the interaction of POPDC proteins with the transport machinery involved in vesicle transport. Moreover, as mentioned before, POPDC proteins interact with the SNARE protein VAMP3, which controls vesicle fusion with the plasma membrane. Thus, it is possible that in response to cAMP binding to POPDC proteins, vesicles are more likely to fuse with the plasma membrane resulting in an enhanced incorporation of the vesicle proteins into the plasma membrane.

### 5.3 The shielding model

The shielding model (Fig. 3C) is a variation of the *switch* model taking into account that POPDC proteins and also some of the interacting proteins are getting phosphorylated in response to βAR activation [54]. It can be envisioned that cAMP binding and phosphorylation of POPDC proteins may lead to conformational changes that affect access of PKA or other kinases to their substrates. Support for such a hypothetical mechanism stems from the electrophysiological measurement of TREK1 current in *Xenopus* oocytes, which is inhibited by cAMP and therefore blunting the effect of co-expression of POPDC proteins [48]. However, TREK-1 current maintained an increased level when the same experiment was performed in the presence of a cAMP binding-deficient POPDC1 mutant. These results may indicate that POPDC proteins form a complex with TREK-1, which may prevent PKA from gaining access and being able to phosphorylate TREK-1.

### 5.4 The sponge model

Finally, the sponge model (Fig. 3D) takes into account that POPDC proteins are abundantly present in the cardiac and skeletal muscle proteome [99], [100]. Assuming a high affinity for cAMP in the range of PKA, which however has thus far only been experimentally determined in case of POPDC1, suggests that a lot of the cellular cyclic nucleotide binding capacity is attributable to POPDC proteins. The sponge model also predicts that in case of a loss of function mutation such as POPDC1^S201F^, which reduces cAMP binding affinity by 50% and also alters the subcellular localization of the mutant protein, or a complete loss of protein expression as in case of the POPDC null mutants, should affect the diffusion rates and compartmentalization of cAMP. In this regard it is noteworthy that recent experimental and computational modelling approaches concluded that although the subcellular localization of adenylate cyclases and phosphodiesterases are important for the creation of cAMP nanodomains, they are by far not sufficient [101], [102]. Several studies have suggested that compartmentalised cAMP signalling requires a slower diffusion rate of cAMP [103] and in this regard POPDC proteins appear to be ideally suited to fulfil this role.

## 6 Outlook

As in any other novel scientific field, many open questions remain to be addressed and it is the firm belief of the authors of this review that we currently are just at the beginning of our understanding of the role of POPDC proteins in cAMP signalling.

An important question that needs to be answered is, whether POPDC proteins are directly interacting with other proteins of the cAMP signalling pathway and whether POPDC proteins modulate the enzymatic activity of these proteins. Thus, we need to address the question, whether POPDC proteins do physically interact with adenylate cyclases or phosphodiesterases. Equally important will be to answer the question, whether there are AKAP proteins, which bind to POPDC proteins. A number of AKAP proteins are localized to the same compartments in cardiac myocytes as POPDC proteins and these candidates need to be tested. It remains to be determined whether PKA or EPAC isoforms show a physical interaction with POPDC proteins and whether interaction may affect their enzymatic activity. It has been shown that POPDC proteins are phosphorylated in response to βAR-dependent phosphorylation [54]. However, whether POPDC proteins and PKA are part of the same signalosome is currently not known. POPDC proteins and HCN channels both bind cAMP and are involved in cardiac pacemaking. Nonetheless, no evidence has been obtained so far for a direct interaction of POPDC1 with HCN4 (the most abundant HCN isoform in the SAN). Patch clamp analysis of I_f_ in SAN myocytes of *Popdc1* null mutants revealed that I_f_ was not affected at baseline or after βAR stimulation [48].

Another important area to look further into is the recent finding of cell proliferation control in cancer cells. In skeletal muscle, the lack of *Popdc1* affects the ability of muscle regeneration [60]. However, currently nothing is known about the role of POPDC genes in cardiac regeneration.

Likewise, we do not understand how POPDC proteins mechanistically affect membrane trafficking of interacting proteins. Thus, the interaction of POPDC proteins with proteins involved in vesicle transport and membrane protein recycling will be an important avenue to be investigated.

Electrophysiological defects have been observed in the heart of patients carrying *POPDC1* mutations, in contrast, structural defects were observed in case of skeletal muscle. It will be interesting to find out, whether mutations in POPDC genes are also able to cause structural defects in the heart such as dilated cardiomyopathy and likewise, whether skeletal muscle could also be functionally affected. We will also need to screen patient populations for the presence of mutations in any of the other POPDC isoforms, given their overlapping expression and function. It is likely that we might find mutations also in these family members.

Currently, no specific drugs are available to activate or inhibit POPDC protein function. Thus, functional analysis is solely dependent on the genetic approach of generating gain- or loss-of-function mutations. Over the years several specific agonists and antagonists have been characterized for PKA, EPAC and HCN channels [104], [105], [106], [107]. None of these compounds have made it into the clinics, however, they are very useful as scientific tools allowing isoform-specific modulation of different effector proteins. At present, similar tools are not available in case of the POPDC protein family. Given the unique sequence of the Popeye domain and in particular of the cyclic nucleotide binding cassette [96], [97], which does not resemble any other cAMP binding domains, it is very likely that specific small molecules could be defined, which will help to analyse the functional involvement of POPDC proteins in different aspects of cAMP signalling. Many more problems will need to be addressed to find out what the *new kids on the block* are up to.
